# Multi-omics integration identifies PGAP3 as a tumor-intrinsic factor associated with CD8^+^ T-cell exclusion in prostate cancer

**DOI:** 10.3389/fmolb.2026.1791456

**Published:** 2026-03-18

**Authors:** Weihao Liu, Guoping Li, Yan Lei, Huixiu Liu, Binhui Wang, Weiming Deng, Yude Hong, Xiangyang Long

**Affiliations:** 1 Department of Urology, The Third Affiliated Hospital of Sun Yat-sen University, Guangzhou, Guangdong, China; 2 Department of Urology, Hainan General Hospital (Hainan Affiliated Hospital of Hainan Medical University), Haikou, Hainan, China; 3 Department of Urology, The Second Affiliated Hospital, University of South China, Hengyang, Hunan, China; 4 Institute of Urology and Organ Transplantation, University of South China, Hengyang, Hunan, China; 5 Hengyang Medical School, University of South China, Hengyang, Hunan, China; 6 Department of Radiology, The Second Affiliated Hospital, University of South China, Hengyang, Hunan, China; 7 Department of Urology, The First Affiliated Hospital, University of South China, Hengyang, Hunan, China

**Keywords:** CD8^+^ T-cell exclusion, Mendelian randomization, multi-omics, PGAP3, prostate cancer, tumor microenvironment

## Abstract

**Background:**

Prostate cancer (PCa) is prototypically immunologically “cold”, characterized by low tumor mutational burden, sparse CD8^+^ T-cell infiltration, and resistance to immune checkpoint blockade. The tumor cell–intrinsic programs driving immune evasion in this context remain incompletely defined.

**Methods:**

We integrated transcriptome-wide Mendelian randomization of PCa GWAS and eQTL data with multi-cohort bulk and single-cell RNA sequencing, spatial transcriptomics, and immune profiling to prioritize candidate genes. Focusing on PGAP3, we characterized its metabolic and immune correlates, and validated the effects of PGAP3 knockdown on proliferation, clonogenicity, and migration in C4-2 and DU145 cells.

**Results:**

PGAP3 was consistently prioritized as a risk gene and was selectively overexpressed in malignant epithelial subpopulations. PGAP3-high cells exhibited increased metabolic activity (biotin, aspartate/asparagine, and sulfur metabolism), coinciding with reduced CXCL14, TNFSF13B, and TNFSF18 expression, lower CD8^+^ T-cell infiltration, and higher immune-exclusion scores. Functionally, PGAP3 silencing significantly impaired proliferation, clonogenic growth, and migration *in vitro*.

**Conclusion:**

Our findings identify PGAP3 as a tumor-intrinsic gene associated with metabolic reprogramming and a CTL/CD8^+^-low immune contexture in PCa, supporting PGAP3 as a potential marker of the immune-cold tumor microenvironment and motivate future mechanistic studies in immunocompetent systems.

## 1 Introduction

Prostate cancer (PCa) is one of the most frequently diagnosed malignancies in men and remains a major cause of cancer-related morbidity and mortality worldwide (Sung et al., 2021; Ju et al., 2023; Siegel et al., 2023). Although advances in screening, imaging and local therapy have improved early detection and disease control, a substantial proportion of patients progress to advanced or metastatic disease with limited durable benefit from current standards of care, including radical prostatectomy, radiotherapy and androgen deprivation therapy (ADT) (Mottet et al., 2021; Chen et al., 2023). These clinical challenges underscore the need to identify new molecular targets and treatment strategies for advanced PCa.

Immune checkpoint inhibitors (ICIs) have transformed the management of several solid tumors; however, their efficacy in PCa has been modest (Venturini and Drake, 2019; Vitkin et al., 2019; Meng et al., 2023; Sooi et al., 2023). PCa is prototypically considered an immunologically “cold” malignancy, characterized by low tumor mutational burden, sparse effector T-cell infiltration and a profoundly immunosuppressive tumor microenvironment (TME). These features contribute to primary resistance to ICIs and other immunotherapies. Understanding how tumor cell–intrinsic programs shape this immune-cold TME is critical for rational target discovery and the development of biomarker-guided immunomodulatory strategies.

The TME, composed of malignant epithelial cells, stromal elements and diverse immune populations, plays a central role in PCa progression and therapeutic resistance (Ayala et al., 2009; Li et al., 2023). Among these components, cytotoxic CD8^+^ T cells are key effectors of anti-tumor immunity, and their density, spatial distribution and functional state have been linked to prognosis and response to immunotherapy (Dorff et al., 2022; Hage Chehade et al., 2024). Nonetheless, the tumor-intrinsic determinants that regulate CD8^+^ T-cell exclusion, dysfunction and immune escape in PCa remain incompletely defined. Identifying such determinants may help convert PCa from an immune-cold to a more immune-responsive disease.

Traditional approaches to therapeutic target discovery have often relied on differential expression and prognostic associations, which are vulnerable to confounding and do not establish causal relationships (Davey Smith and Hemani, 2014; Daghlas and Gill, 2023). Mendelian randomization (MR), by leveraging genetic variants as instrumental variables, provides a powerful framework to infer putative causal links between gene expression and disease risk using genome-wide association study (GWAS) and expression quantitative trait loci (eQTL) data (Porcu et al., 2019; Gleason et al., 2021; Daghlas and Gill, 2023). Recent studies have begun to apply MR to prioritize candidate targets across cancers (Liu et al., 2020; Robinson et al., 2021), but its integration with single-cell and spatial transcriptomics to dissect the PCa TME and immune landscape remains limited.

Post-GPI attachment to proteins phospholipase 3 (PGAP3), located on chromosome 17q12, encodes a glycosylphosphatidylinositol (GPI)-specific phospholipase involved in GPI-anchor remodeling (Maeda et al., 2007; Altassan et al., 2023). PGAP3 has been implicated in several malignancies, including gastric, cervical, pancreatic and breast cancers, where altered PGAP3 expression or splicing has been associated with tumor progression and immune regulation (Liu et al., 2020; Jun et al., 2021; Wang et al., 2023; Tan et al., 2024). PGAP3 expression has also been linked to lymph node metastasis and decreased CD8^+^ T-cell infiltration in breast cancer, and PGAP3 deficiency enhances T-cell receptor signaling in mouse models, suggesting a role in modulating anti-tumor immunity (Murakami et al., 2012; Hao et al., 2023). However, the specific contributions of PGAP3 to PCa biology and the PCa TME are largely unknown.

In this study, we implemented an integrative multi-omics framework combining transcriptome-wide MR and summary-based MR of PCa GWAS with blood and prostate eQTLs, multi-cohort bulk RNA sequencing, single-cell RNA sequencing, spatial transcriptomics and comprehensive immune profiling to prioritize and characterize candidate genes in PCa. Through this pipeline, we identified PGAP3 as a putative PCa risk gene selectively overexpressed in malignant epithelial subpopulations. We then systematically examined the metabolic and immune correlations of PGAP3, and its association with CD8^+^ T-cell infiltration and features of immune evasion. Finally, we performed siRNA-mediated loss-of-function assays in PCa cell lines to validate the impact of PGAP3 on malignant phenotypes. Our findings highlight PGAP3 as a tumor-intrinsic candidate associated with the immune-cold TME in PCa and support its potential as a biomarker and therapeutic target.

## 2 Materials and methods

### 2.1 Genetic and transcriptomic datasets

Cis-expression quantitative trait loci (cis-eQTL) summary statistics were obtained from the eQTLGen Consortium (16,987 genes; 31,684 cis-eQTLs) based on whole-blood samples from predominantly healthy European participants (Võsa et al., 2021). For transcriptome-wide Mendelian randomization (TWMR), we restricted cis-eQTLs to SNPs located within 5 kb upstream or downstream of druggable genes, yielding 2,554 genes for downstream analyses. Prostate tissue–specific cis-eQTLs were additionally obtained from the GTEx (v8) project to investigate tissue-specific regulation (GTEx Consortium, 2020).

Prostate cancer (PCa) GWAS summary statistics for the discovery stage were derived from a large multi-ancestry meta-analysis including 122,188 PCa cases and 604,640 controls of European ancestry (ID: GCST90274714) (Hao et al., 2023). FinnGen Release 10 (ID: finngen_R10_C3_PROSTATE_EXALLC; 15,199 cases and 131,266 controls) served as an independent replication cohort (Kurki et al., 2023).

Bulk RNA-sequencing data and corresponding clinical information for PCa were downloaded from TCGA-PRAD using the “TCGAbiolinks” R package (Colaprico et al., 2016). Raw counts were converted to transcripts per million (TPM) and log2-transformed for downstream analyses. An external GEO dataset (GSE97284) containing primary tumor and benign prostate samples was used as an independent cohort and processed in the same way.

Single-cell RNA-seq (scRNA-seq) data from primary PCa (GSE181294; 18 primary tumors) (Hirz et al., 2023) and spatial transcriptomic (ST) data (GSE230282) (Cable et al., 2022) were used to characterize cell states and the spatial tumor microenvironment.

### 2.2 Mendelian randomization and gene prioritization

Two-sample MR was performed using the TwoSampleMR R package (version 0.6.6) (Hemani et al., 2018). Exposure (gene expression) and outcome (PCa risk) summary statistics were harmonized to ensure alignment of alleles and effect directions. Genetic instruments were required to satisfy the core MR assumptions (Davies et al., 2018): (1) SNPs strongly associated with the exposure (P < 5 × 10^−8^); (2) no association with confounders of the exposure–outcome relationship; and (3) effects on the outcome only through the exposure. To minimize population stratification, analyses were restricted to individuals of European ancestry, and instruments with an F-statistic <10 were excluded (Palmer et al., 2012). Linkage disequilibrium (LD) clumping was performed using a 1,000 Genomes European reference panel (*r* ^2^ < 0.001) to ensure independence among SNPs (Abecasis et al., 2012).

Five MR estimators were applied: inverse-variance weighted (IVW), MR-Egger, weighted median, simple mode and weighted mode. IVW was used as the primary method (Burgess et al., 2019), and for genes with multiple instruments, the MR-Egger intercept and Cochran’s Q tests were used to assess horizontal pleiotropy and heterogeneity (Greco et al., 2015). In the discovery cohort, genes with IVW P < 0.05 and no evidence of horizontal pleiotropy were retained; genes showing consistent effect directions in FinnGen were considered TMR-supported candidates.

To incorporate prostate tissue–specific regulation, we further performed summary-data–based MR (SMR) (Zhu et al., 2016) using GTEx prostate cis-eQTL data as exposures and PCa GWAS (GCST90274714 and FinnGen) as outcomes. For each gene, the top cis-eQTL SNP (P < 1 × 10^−5^) was selected as the instrument. The HEIDI test implemented in SMR was used to distinguish pleiotropy from LD; associations with P_SMR < 0.05 and P_HEIDI > 0.05 were considered consistent with a shared causal variant. Only protein-coding genes meeting these criteria in at least one cohort were retained. Genes supported by both TMR and SMR were defined as robust PCa risk genes, among which VAMP3 and PGAP3 showed risk-increasing effects and were prioritized for downstream analyses.

### 2.3 Single-cell, spatial and pathway analyses

Raw scRNA-seq matrices (GSE181294) were processed with Seurat. Quality control and cell-type annotation were performed in strict accordance with the original publication (Hirz et al., 2023), utilizing their validated metadata to distinguish malignant luminal cells. To define the “PGAP3^+^ malignant” subset, we used a threshold of detectable expression (read counts >0) to account for data sparsity, classifying cells with non-zero counts as positive. Batch effects across samples were corrected using a Harmony-based integration workflow, followed by dimensionality reduction with UMAP and clustering with the Louvain algorithm. Differentially expressed genes between malignant subpopulations were identified using the FindMarkers function with |log_2_ fold-change| ≥ 0.5.

ST data (GSE230282) were normalized and processed using SCTransform in Seurat. Spatial expression patterns were visualized with Seurat, and the RCTD algorithm was applied to deconvolute spatial spots and infer cell-type compositions using scRNA-defined cell types as references, thereby linking single-cell and spatial profiles (Cable et al., 2022). Spatial proximity between PGAP3-high malignant regions and immune signals was quantified using a k-nearest-neighbor (k = 6) neighborhood statistic and a nearest-neighbor distance statistic. Statistical significance was assessed by permutation tests (B = 10,000) that shuffled immune signal values (or binary high/low labels) across spots while preserving spatial coordinates; empirical p-values were reported using a +1 correction.

Metabolic pathway activity at the single-cell level was quantified using the scMetabolism package, which computes pathway scores based on single-sample gene set enrichment analysis (ssGSEA) (Wu et al., 2022). Metabolic scores were compared between cell populations, with particular focus on differences between PGAP3^+^ and PGAP3^-^ malignant epithelial cells.

Cell–cell communication networks among TME cell types were inferred using CellChat (Jin et al., 2021) and the “CellChatDB.human” ligand–receptor database, focusing on signaling between PGAP3^+^ malignant epithelial cells and cytotoxic T lymphocytes. Tumor cell developmental trajectories and progression vectors were reconstructed using the VECTOR tool to infer pseudotime and developmental directions of malignant subpopulations (Zhang et al., 2020), and to assess how PGAP3 expression varied along these trajectories.

To evaluate PGAP3 at the protein and genetic levels, immunohistochemistry and immunofluorescence data were obtained from the Human Protein Atlas (HPA; https://www.proteinatlas.org; version 24.0) (Uhlén et al., 2015) to assess PGAP3 expression patterns and subcellular localization in prostate and other tissues. A phenome-wide association study (PheWAS) at the PGAP3 locus was performed using the AstraZeneca PheWAS Portal (Wang et al., 2021), and survival associations for the top PGAP3 SNP were explored using the SUrvival related cancer Multi-omics database via MEndelian Randomization (SUMMER) (Xin et al., 2023).

Disease Ontology (DO), Gene Ontology (GO) and Kyoto Encyclopedia of Genes and Genomes (KEGG) enrichment analyses were conducted using the clusterProfiler R package (Yu et al., 2012). Gene set enrichment analysis (GSEA) was used to identify signaling and metabolic pathways enriched in PGAP3-high malignant cells. Marker genes of the “PGAP3^+^ Malignant” subgroup were used to construct diagnostic and prognostic models: univariate logistic regression and LASSO were applied to build multiple classification models (logistic regression, linear discriminant analysis, support vector machine, naive Bayes, k-nearest neighbors, decision tree and random forest), with TCGA-PRAD as the training set and GSE97284 as an external validation cohort; a LASSO–Cox model was used to derive a prognostic risk score, evaluated by Kaplan–Meier and time-dependent ROC analyses.

### 2.4 Immune infiltration analyses

To examine immune infiltration across cancers, TIMER 2.0 was used to estimate CD8^+^ T-cell abundance and correlate it with PGAP3 expression (Li et al., 2017). In TCGA-PRAD, the ESTIMATE algorithm was applied to derive immune, stromal and ESTIMATE scores and tumor purity (Yoshihara et al., 2013). For group-wise comparisons, TCGA-PRAD tumors were dichotomized into PGAP3-high and PGAP3-low groups using the median PGAP3 expression as the cut-off (50th percentile), given that no established clinical threshold is available. PCa samples were dichotomized into PGAP3-high and PGAP3-low groups to compare immune features, including expression of the chemokine CXCL14 and immune stimulators TNFSF13B and TNFSF18.

T-cell functional states were evaluated using the T cell state identifier (TCellSI) algorithm (Yang et al., 2024), which infers eight T-cell states from bulk transcriptomes based on predefined marker gene sets and a Mann–Whitney U statistic–based scoring scheme. TCellSI-derived state scores were used to assess associations between PGAP3 expression and diverse T-cell functional states.

### 2.5 Cell culture and *in vitro* functional assays

The cell lines present in this study (C4-2, DU145 and RWPE-1) were obtained from the Cell Bank of the Chinese Academy of Sciences (Shanghai, China). C4-2 and DU145 cells were cultured in RPMI-1640 medium supplemented with 10% fetal bovine serum (FBS) and 1% penicillin–streptomycin. RWPE-1 cells were grown in Keratinocyte-SFM supplemented with bovine pituitary extract and recombinant epidermal growth factor. All cell lines were incubated at 37 °C in a humidified atmosphere with 5% CO_2_.

A siRNA targeting PGAP3 (siPGAP3-189) was adopted from a published study (Wang et al., 2023). The siRNA sequences are listed in Supplementary Table S16. For transfection, 3 × 10^3^ cells per well were seeded into 24-well plates in 500 µL RPMI-1640 and transfected with siPGAP3 or negative control siRNA (siNC) using Lipofectamine RNAiMAX (Thermo Fisher Scientific, 13778030) according to the manufacturer’s instructions. Cells were harvested 48–72 h after transfection for knockdown validation and functional assays.

Total RNA was extracted using TRIzol reagent (Invitrogen, 66003), and first-strand cDNA was synthesized using a commercial cDNA synthesis kit (Vazyme, R323-01). Quantitative real-time PCR (qRT-PCR) was performed with SYBR Green chemistry (Vazyme, Q712-02). Relative mRNA expression levels were calculated using the 2^−^ΔΔCt method with GAPDH as the internal control. Primer sequences are provided in Supplementary Table S17.

For western blotting, cells were lysed in RIPA buffer supplemented with protease inhibitors, and equal amounts of protein were separated by SDS–PAGE and transferred onto PVDF membranes. Membranes were blocked with 5% non-fat milk and incubated with primary antibodies against PGAP3 (Immunoway, YN5206) and β-actin (Affinity, AF7018), followed by HRP-conjugated secondary antibodies (Cell Signaling Technology, 7074S). Protein bands were visualized using enhanced chemiluminescence (NCM, P10100).

Cell proliferation was assessed using the CCK-8 assay (Dojindo, CK04). Briefly, siRNA-transfected cells were seeded into 96-well plates, incubated with CCK-8 solution at indicated time points, and absorbance at 450 nm was measured. Clonogenic capacity was evaluated by colony formation assays: 1 × 10^4^ transfected cells were seeded into 6-well plates, cultured for approximately 2 weeks, then fixed, stained with crystal violet, imaged and counted.

Cell migration was assessed by wound-healing and Transwell assays. For wound-healing assays, confluent monolayers were scratched with a 200-µL pipette tip, washed with PBS, and cultured in 1% FBS medium; images were acquired at 0 h and 24 h, and wound closure was quantified using ImageJ. For Transwell migration assays, cells were seeded in serum-free medium in the upper chambers of Transwell inserts, with medium containing 10% FBS in the lower chambers as chemoattractant. After incubation, migrated cells on the lower surface were fixed with 4% paraformaldehyde, stained with crystal violet, imaged and counted.

### 2.6 Statistical analysis

Bioinformatic analyses were performed using R software (version 4.4.2). Spearman’s correlation was used for correlation analysis. Continuous variables with normal distributions were analyzed using Student’s t-test, whereas the Mann–Whitney U-test was applied to those with non-normal distributions. For *in vitro* experiments, data are presented as mean ± SD from at least three independent experiments. Two-group comparisons were performed using two-tailed Student’s t-test, and one-way ANOVA was applied where appropriate. Two-tailed P values <0.05 were considered statistically significant. Detailed information regarding the specific versions of all software and R packages used in this study is provided in Supplementary Table S18.

## 3 Results

### 3.1 Identification of prostate cancer risk genes using mendelian randomization

To identify genes whose expression may exert a putative causal effect on PCa risk, we performed TMR using cis-eQTLs as instruments for genetically proxied gene expression, which are less prone to confounding than conventional expression–outcome associations.

**FIGURE 1:**
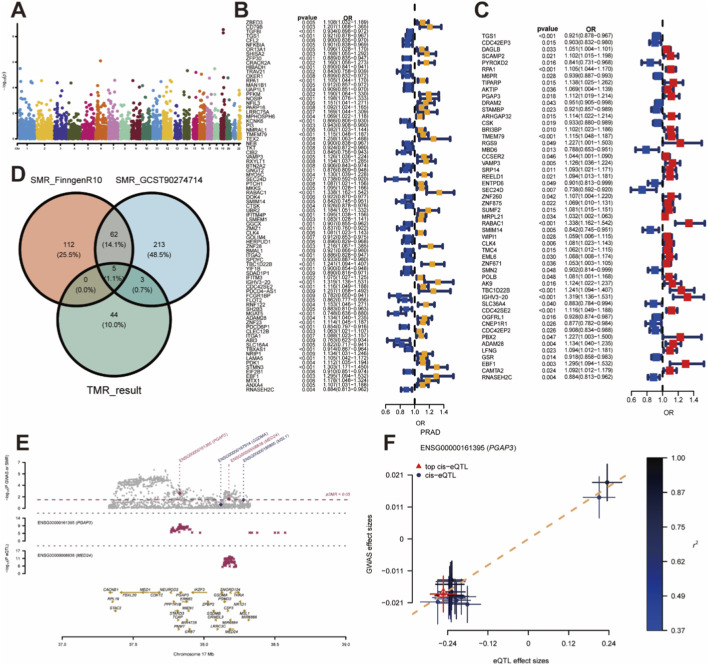
Selection of Prostate Cancer Risk Genes by MR. **(A)** Manhattan plot of transcriptome-wide MR (TMR) results in the discovery phase (top 100 genes shown). **(B)** Forest plots of the replication phase results for genes with IVW P < 0.01. **(C)** Forest plots for genes showing significant associations (P < 0.05) in both the discovery and replication phases. **(D)** Intersection of TMR-validated genes with SMR-identified genes yielding five robust candidate genes. **(E,F)** SMR locus plots for PGAP3.

In the discovery stage, we leveraged summary statistics from a large multi-ancestry GWAS of prostate cancer (GCST90274714; 122,188 European-ancestry cases and 604,640 controls). After excluding genes with evidence of horizontal pleiotropy, TMR identified genetically predicted expression of 507 genes associated with PCa risk (Figure 1A; Supplementary Tables S1–S3). In the replication stage, we applied the same TMR pipeline to the FinnGen R10 prostate cancer GWAS (15,199 cases and 131,266 controls), identifying 326 genes associated with PCa risk (Figure 1B; Supplementary Tables S4–S6). A total of 52 genes showed consistent direction and magnitude of effect across both stages and were retained for further analysis (Figure 1C; Supplementary Table S7).

To further refine and validate candidate genes in a tissue-specific manner, we conducted summary-data-based Mendelian randomization (SMR) using prostate cis-eQTL data from GTEx as the exposure and the GCST90274714 and FinnGen cohorts as outcomes. SMR identified 151 and 97 genes, respectively, whose prostate expression was associated with PCa risk and passed the HEIDI test (p_SMR < 0.05 and p_HEIDI > 0.05; Supplementary Tables S8–S9).

Intersecting the 52 TMR-validated genes with the two prostate SMR gene sets yielded five robust candidate genes—VAMP3, DRAM2, RNASEH2C, PYROXD2, and PGAP3 (Figure 1D; Table 1). Among these, VAMP3 and PGAP3 showed risk-increasing effects, whereas DRAM2 and RNASEH2C were protective, and PYROXD2 displayed discordant effects between TMR and SMR analyses (Table 1). On this basis, VAMP3 and particularly PGAP3 were prioritized for in-depth multi-omics characterization in subsequent analyses (Figures 1E,F).

**TABLE 1:** Five candidate genes from TMR and SMR results.

Method	Cohort	Metrics	PGAP3	VAMP3	DRAM2	RNASEH2C	PYROXD2
TMR	GCST90274714	OR	1.048	1.037	0.966	0.964	0.944
		pval	0.009	0.009	0.001	0.047	0.046
		Pleiotropy_pval	0.192	0.525	0.353	0.686	0.596
		Heterogeneity_pval	0.463	0.366	0.448	0.119	0.237
	FinnGenR10	OR	1.112	1.126	0.951	0.884	0.841
		pval	0.018	0.005	0.043	0.004	0.016
		Pleiotropy_pval	0.194	0.341	0.434	0.463	0.436
		Heterogeneity_pval	0.392	0.374	0.633	0.266	0.383
SMR	GCST90274714	topSNP	rs2952152	rs1048785	rs3762374	rs11227248	rs7913541
		b_SMR	0.072	0.054	−0.028	−0.066	0.041
		p_SMR	0.005	0.038	0.004	0.012	0.044
		p_HEIDI	0.678	0.258	0.454	0.856	0.898
	FinnGenR10	topSNP	rs2952152	rs1048785	rs3762374	rs11227248	rs7913541
		b_SMR	0.157	0.160	−0.070	−0.241	0.189
		p_SMR	0.011	0.005	0.003	0.001	0.001
		p_HEIDI	0.173	0.582	0.456	0.075	0.339

### 3.2 PGAP3 is selectively expressed in malignant epithelial cells and associated with PCa progression and prognosis

**FIGURE 2:**
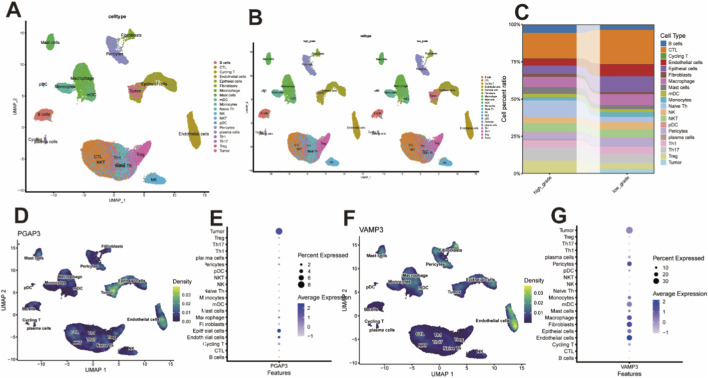
Single-cell expression profiling for PCa. **(A,B)** UMAP Plot categorizing single-cell data by cell types and grade. **(C)** Composite plot showing the composition of cell sources and cell types. **(D–G)** DensityPlot and DotPlot of PGAP3 and VAMP3, respectively.

To gain cellular-resolution insight into the high-risk biomarkers identified by MR, we analyzed single-cell RNA-sequencing (scRNA-seq) data from low-grade and high-grade PCa. The integrated analysis revealed a diverse tumor microenvironment comprising 20 distinct cell types (Figure 2A). The relative abundance of multiple cell populations differed markedly between low- and high-grade tumors (Figures 2B,C), indicating extensive remodeling of the tumor ecosystem during PCa progression. Among the two MR-prioritized genes, VAMP3 and PGAP3, we focused on PGAP3 because it showed more tumor cell–restricted expression, with pronounced enrichment in malignant epithelial clusters (Figures 2D–G), suggesting a potential tumor cell–intrinsic role in PCa.

**FIGURE 3:**
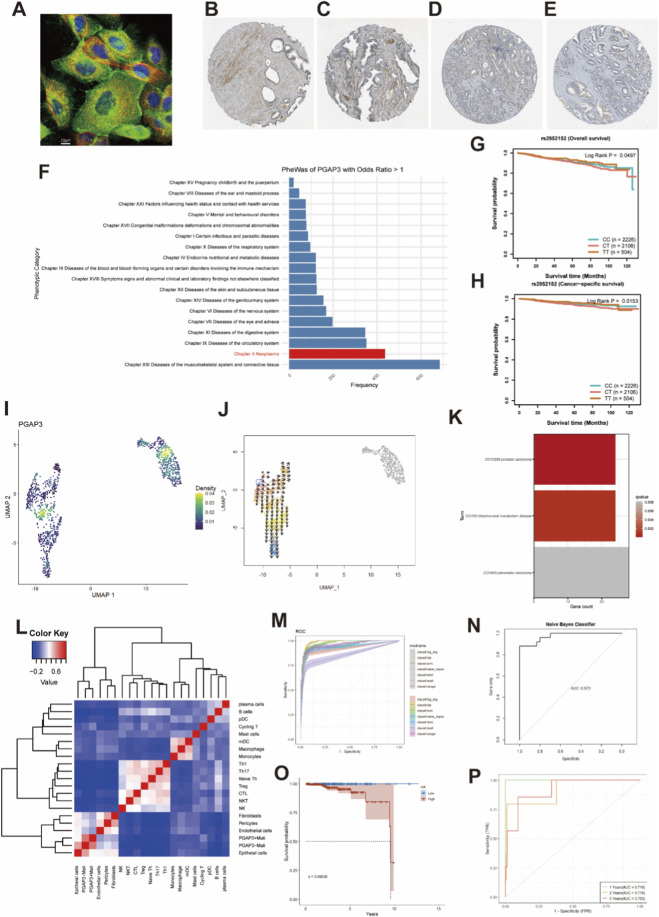
PGAP3 is associated with PCa progression and prognosis. **(A)** Immunofluorescence imaging of PGAP3. The green fluorescence represents PGAP3 protein, the blue fluorescence denotes the nucleus, and the red fluorescence corresponds to microtubules. Image sourced from HPA database. **(B)** Immunohistochemical staining of normal prostate tissue from HPA database (patient id: 2053, https://images.proteinatlas.org/16591/39885_A_3_5.jpg). **(C)** Immunohistochemical staining of normal prostate tissue from HPA database (patient id: 2098, https://images.proteinatlas.org/16591/39885_A_2_5.jpg). **(D)** Immunohistochemical staining of PCa tissue from HPA database (patient id: 3,580, https://images.proteinatlas.org/16591/39882_A_8_5.jpg). **(E)** Immunohistochemical staining of PCa tissue from HPA database (patient id: 3,577, https://images.proteinatlas.org/16591/39882_A_9_3.jpg). **(F)** Traits Significantly associated with PGAP3 using AstraZeneca PheWAS portal. **(G,H)** KM plot for PGAP3’s topSNP from SUMMER database; Patients are stratified by their genotype: CC (homozygous for the major allele), CT (heterozygous), and TT (homozygous for the minor allele). **(I)** DensityPlot of PGAP3 in tumor cell type. **(J)** The vector representation of developmental directions for tumor cells. **(K)** DO enrichment analysis for marker genes in the ‘PGAP3+ Malignant’ subgroup. **(L)** Heatmap of cellular subpopulation correlations. **(M)** ROC curves for the diagnostic model evaluated across seven machine learning algorithms in the training set. **(N)** ROC curve for the diagnostic model utilizing the Naive Bayes Classifier algorithm in the validation set. **(O,P)** Kaplan-Meier **(K–M)** and ROC curves for the prognostic model in the TCGA cohort.

To further characterize PGAP3 at the protein level, we examined data from the Human Protein Atlas. Immunofluorescence imaging demonstrated that PGAP3 is predominantly localized in the cytoplasm (Figure 3A). Consistently, immunohistochemistry (IHC) revealed stronger PGAP3 staining in malignant prostatic epithelium than in normal epithelium (Figures 3B–E), supporting upregulation of PGAP3 in PCa tissues. We next conducted a phenome-wide association study (PheWAS) at the PGAP3 locus using 17,361 dichotomous phenotypes and 1,419 quantitative traits from the AstraZeneca PheWAS Portal. The spectrum of traits associated with PGAP3 (odds ratio > 1) showed that tumor-related phenotypes represented one of the most frequent categories, suggesting that genetically influenced PGAP3 variation is recurrently linked to cancer-related traits, including those relevant to PCa progression (Figure 3F; Supplementary Table S10). In addition, the top PGAP3 SNP, rs2952152, was significantly associated with both overall and cancer-specific survival in PCa, indicating potential prognostic relevance at the germline level (Figures 3G,H).

Single-cell analysis also revealed marked heterogeneity of PGAP3 expression within the malignant epithelial compartment (Figure 3I). Using the VECTOR algorithm, we inferred developmental trajectories across tumor cell subpopulations and identified putative progenitor-like states and progression vectors along which PGAP3 expression varied (Figure 3J). Given this heterogeneity, we stratified malignant cells into PGAP3^+^ malignant and PGAP3^-^ malignant groups based on detectable PGAP3 expression. Correlation analysis of cell-type compositions showed that PGAP3^-^ malignant cells exhibited stronger similarity to normal epithelial cells, whereas PGAP3^+^ malignant cells formed a more distinct tumor-associated compartment (Figure 3L). Disease Ontology (DO) enrichment of marker genes from the PGAP3^+^ malignant subgroup demonstrated significant enrichment for PCa and other neoplastic terms (Figure 3K; Supplementary Table S11), further supporting that the PGAP3^+^ malignant program is closely linked to prostate tumor biology.

We then evaluated the clinical utility of PGAP3-related transcriptional programs by constructing diagnostic and prognostic models based on marker genes of the PGAP3^+^ malignant subgroup. For diagnosis, we built binary classification models using seven machine learning algorithms. These models achieved high discriminative performance in the training set (TCGA cohort) and retained robust diagnostic accuracy in an independent GEO validation cohort (Figures 3M,N; Supplementary Figure S1). For prognosis, a LASSO–Cox regression model derived from PGAP3^+^ malignant marker genes yielded a risk score that effectively stratified patients by overall survival in the TCGA cohort, with clear separation of Kaplan–Meier survival curves and favorable time-dependent ROC characteristics (Figures 3O,P). Together, these findings indicate that PGAP3 is selectively upregulated in malignant epithelial cells and that PGAP3-associated transcriptional signatures carry substantial diagnostic and prognostic information in PCa.

### 3.3 PGAP3-associated metabolic reprogramming and immune exclusion in PCa

**FIGURE 4:**
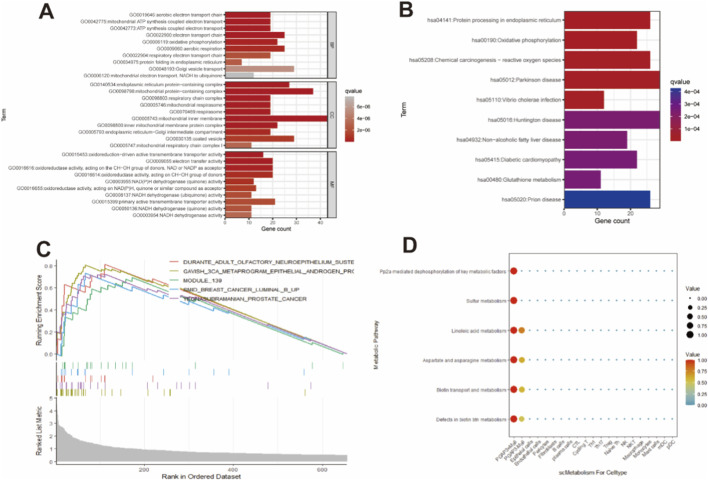
The role of PGAP3 in special or unique metabolic pathways. **(A–C)** Enrichment analysis results of GO, KEGG and GSEA for marker genes in the ‘PGAP3+ Malignant’ subgroup. **(D)** scMetabolism results for ‘PGAP3+ Malignant’ subgroup.

To investigate molecular programs associated with PGAP3 in malignant epithelial cells, we performed Gene Ontology (GO), Kyoto Encyclopedia of Genes and Genomes (KEGG) and gene set enrichment analysis (GSEA) of marker genes upregulated in the PGAP3^+^ malignant subgroup. These analyses showed that PGAP3^+^ malignant cells were significantly enriched for multiple metabolic pathways (Figures 4A–C; Supplementary Tables S12–S14). Consistently, single-cell metabolic profiling using scMetabolism revealed globally higher metabolic activity scores in PGAP3^+^ malignant cells compared with PGAP3^-^ malignant cells (Figure 4D). PGAP3^+^ malignant cells displayed a distinctive metabolic pattern characterized by enrichment of biotin metabolism, aspartate and asparagine metabolism, linoleic acid metabolism, sulfur metabolism and PP2A-related pathways (Figure 4D; Supplementary Figure S3), indicating that PGAP3-high tumor cells are coupled to specific metabolic reprogramming programs in PCa.

**FIGURE 5:**
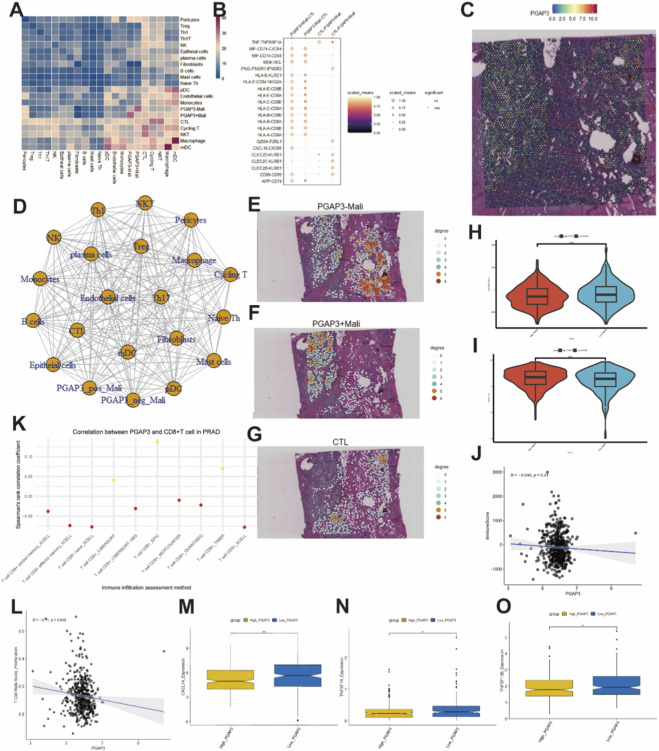
The interplay between PGAP3 and T cells. **(A)** CellChat analysis results of intercellular communication among various cellular subpopulations. **(B)** The communication patterns and potential signaling pathways involved in the crosstalk between the ‘PGAP3+ Malignant’ subgroup and CTL subgroup. **(C)** Spatial distribution of PGAP3 in PCa tissue. **(D)** Cellular interaction network among various subpopulations as inferred from spatial transcriptomics analysis. **(E–G)** Spatial distribution of ‘PGAP3-Malignant’, ‘PGAP3 + Malignant’, ‘CTL’ in PCa tissue. **(H–J)** ESTIMATE analysis results of the immune scores and tumor purity. **(K)** Spearman’s correlation scatter plots for PGAP3 expression and CD8^+^ T cell infiltration across various cancer types from the TIMER2.0 database. **(L)** Scatter plots illustrating the correlation between PGAP3 expression and T cell states as evaluated by the TCellSI algorithm. **(M–O)** Box plots comparing the gene expression levels of CXCL14, TNFSF18, and TNFSF13B between high and low PGAP3 expression groups.

Given prior reports linking PGAP3 to lymph node metastasis and reduced CD8^+^ T-cell infiltration in breast cancer, as well as enhanced T-cell receptor signaling in PGAP3-deficient mice (Raghupathy et al., 2015; Hao et al., 2023), we next examined how PGAP3 expression relates to T-cell–associated features in the PCa tumor microenvironment. Using CellChat to infer intercellular communication networks, we observed distinct patterns of ligand–receptor signaling between cytotoxic T lymphocytes (CTLs) and PGAP3^+^ versus PGAP3^-^ malignant cells (Figures 5A,B), suggesting altered crosstalk between tumor cells and CTLs in PGAP3-high states.

To assess spatial relationships between PGAP3-expressing tumor cells and CD8^+^ T cells *in situ*, we analyzed spatial transcriptomic data from PCa tissue sections. To clearly characterize the immune landscape, we adopted a specific operational definition for ‘immune exclusion’: the spatial restriction of CD8^+^ T cells, particularly the cytotoxic subset (CTLs), to the peritumoral stroma with minimal infiltration into tumor nests. This contrasts with an ‘immune desert’ phenotype, where T cells are scarce in both the tumor and stroma. PGAP3^+^ and PGAP3^-^ malignant spots showed clearly different spatial distributions, further supporting intratumoral heterogeneity of PGAP3 expression (Figures 5C–F). Notably, PGAP3^+^ malignant–enriched regions showed reduced local CTL signal and increased spatial separation from CTL-high spots (Figures 5F,G; Supplementary Figures S4A,B), as supported by permutation-based neighborhood and distance analyses.

We then validated these observations using bulk RNA-seq data. In TCGA-PRAD, PGAP3-high tumors had significantly lower ESTIMATE scores and higher tumor purity (Figures 5H–J). ImmuneScore showed a weak negative trend with PGAP3 but did not reach statistical significance (Figure 5J). To identify the immune subset driving this pattern, we next used TIMER 2.0 deconvolution, which showed that PGAP3 expression was negatively correlated with CD8^+^ T-cell infiltration in seven of ten deconvolution methods (Figure 5K; Supplementary Table S16). Using the TCellSI algorithm, PGAP3 expression also showed statistically significant but weak negative associations with several T-cell functional states (with the exception of senescence; Figure 5L; Supplementary Figures S4C–J), suggesting a subtle association between PGAP3 and T-cell functional state signatures.

To explore potential mechanisms underlying reduced CD8^+^ T-cell infiltration, we compared the expression of chemokines and immune-stimulatory molecules between PGAP3-high and PGAP3-low PCa samples. High PGAP3 expression was associated with significantly lower levels of the chemokine CXCL14 and immune stimulators TNFSF13B and TNFSF18 (Figures 5M–O), molecules known to support T-cell recruitment and activation. These findings suggest that PGAP3-high tumors are characterized by both altered metabolic programs and diminished expression of key immune-activating factors, coinciding with reduced CD8^+^ T-cell infiltration and features of immune exclusion in PCa.

### 3.4 Functional validation of PGAP3 in PCa cells

**FIGURE 6:**
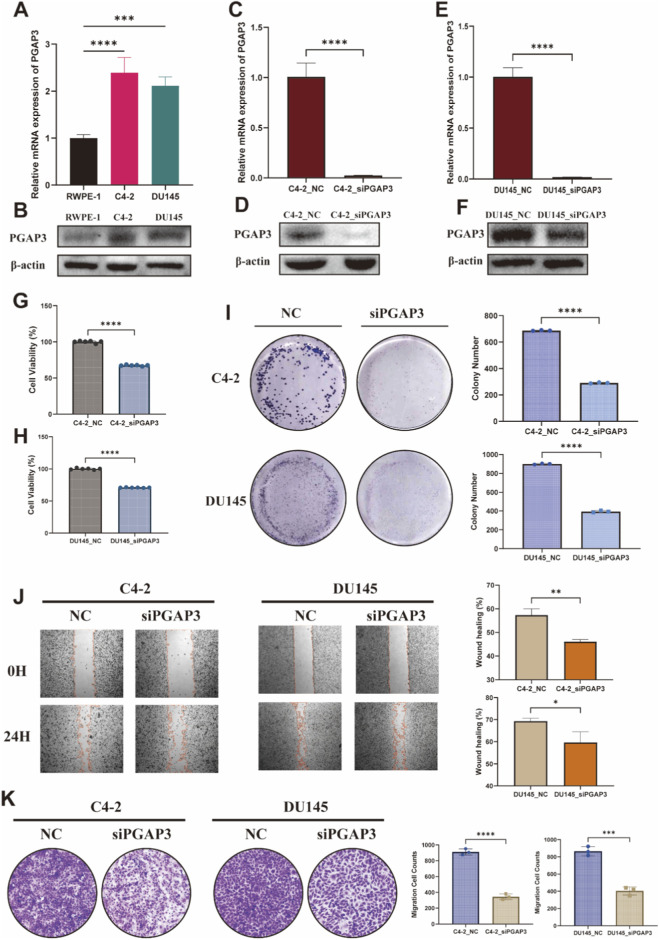
*In vitro* validation of PGAP3 in PCa cell lines. **(A,B)** qRT-PCR and Western blot showing PGAP3 expression in RWPE-1, C4-2 and DU145. **(C–F)** Knockdown efficiency of PGAP3 in C4-2 and DU145 cells transfected with siPGAP3 and siNC as determined by qRT-PCR and Western blot. **(G,H)** CCK-8 assays for cell proliferation after PGAP3 silencing in C4-2 and DU145. **(I)** Colony formation assay and quantification after PGAP3 silencing in C4-2 and DU145 cells. **(J)** Wound healing assay under low-serum conditions and quantification in C4-2 and DU145 cells. **(K)** Transwell migration assay and quantification in C4-2 and DU145 cells. Data are presented as mean ± SD. All quantitative results represent at least three independent biological replicates. Statistical significance was determined using Student’s t-test. *P < 0.05, **P < 0.01, *P < 0.001.

To experimentally assess the role of PGAP3 suggested by our integrative omics analyses, we first examined PGAP3 expression in prostate epithelial and cancer cell lines. qRT-PCR showed that PGAP3 mRNA levels were markedly higher in the prostate cancer cell lines C4-2 and DU145 than in the non-malignant prostate epithelial cell line RWPE-1 (Figure 6A). Consistently, western blotting confirmed increased PGAP3 protein abundance in C4-2 and DU145 cells relative to RWPE-1 (Figure 6B), indicating aberrant upregulation of PGAP3 in prostate cancer cell models.

We next transiently silenced PGAP3 using a published siRNA (siPGAP3), with a non-targeting siRNA serving as the negative control (siNC). Efficient PGAP3 knockdown was verified at both the mRNA and protein levels by qRT-PCR and western blotting, respectively, in C4-2 and DU145 cells (Figures 6C–F), confirming robust depletion of PGAP3 suitable for subsequent functional assays.

Functionally, PGAP3 silencing significantly impaired cell growth. In CCK-8 assays, siPGAP3-transfected C4-2 and DU145 cells exhibited reduced proliferation compared with siNC controls (Figures 6G,H). In line with this, colony formation assays demonstrated that PGAP3 depletion decreased clonogenic potential, as evidenced by fewer and smaller colonies in the siPGAP3 group than in the siNC group for both cell lines (Figure 6I). These findings indicate that PGAP3 supports proliferative and clonogenic capacities in PCa cells.

Because differences in proliferation can confound migration measurements, we assessed cell motility under low-serum conditions. In wound-healing assays, PGAP3-depleted C4-2 and DU145 cells showed delayed wound closure relative to siNC controls (Figure 6J), indicating reduced migratory capability. Consistently, Transwell migration assays revealed that the number of migrated cells was significantly decreased upon PGAP3 knockdown compared with siNC in both cell lines (Figure 6K).

Taken together, these *in vitro* experiments demonstrate that PGAP3 is upregulated in prostate cancer cell lines and that transient PGAP3 silencing suppresses proliferation, clonogenic growth and migration. These functional data support a role for PGAP3 in promoting malignant phenotypes of PCa cells, providing experimental reinforcement for the associations identified in our multi-omics analyses.

## 4 Discussion

Mendelian randomization (MR) uses germline genetic variants as instrumental variables to infer putative causal relationships between molecular traits and disease outcomes based on genome-wide association study (GWAS) data (Birney, 2022). Compared with traditional approaches that prioritize therapeutic targets solely based on overexpression or prognostic associations, MR offers greater protection against confounding and reverse causation (Georgakis and Gill, 2021; Larsson et al., 2023; Papadopoulou et al., 2023; Zeitoun and El-Sohemy, 2023). In this study, we integrated transcriptome-wide MR (TMR) with summary-data–based MR (SMR) in a two-stage design, using blood and prostate cis-eQTLs together with large PCa GWAS datasets, and then combined these results with bulk, single-cell and spatial transcriptomics. This multi-step cross-validation strategy identified five robust candidate genes associated with PCa risk, among which VAMP3 and PGAP3 showed risk-increasing effects. Single-cell analysis further revealed that PGAP3 is selectively enriched in malignant epithelial cell populations, leading us to prioritize PGAP3 for in-depth characterization.

PGAP3, located on chromosome 17q12, encodes a glycosylphosphatidylinositol (GPI)-specific phospholipase involved in the remodeling of GPI-anchored proteins within the Golgi apparatus (Altassan et al., 2023; Wang et al., 2023). Previous work has implicated PGAP3 and PGAP3-related transcripts in several malignancies, including gastric, cervical, pancreatic and breast cancers, where altered PGAP3 expression, co-amplification with ERBB2, alternative splicing or circRNA isoforms have been associated with tumor progression and immune regulation (He et al., 2020; Jun et al., 2021; Liu et al., 2023; Wang et al., 2023). High PGAP3 expression in breast cancer has been linked to lymph node metastasis and reduced CD8^+^ T-cell infiltration, and PGAP3 deficiency in mice has been shown to enhance T-cell receptor signaling, suggesting that PGAP3 participates in the regulation of anti-tumor immunity (Raghupathy et al., 2015; Hao et al., 2023). Our findings extend these observations to PCa by showing that PGAP3 is upregulated in malignant epithelial subpopulations, associated with metabolic reprogramming, reduced CD8^+^ T-cell abundance and features of immune evasion in the PCa tumor microenvironment (TME), and functionally required for proliferative and migratory phenotypes in PCa cell lines.

In PCa, PGAP3^+^ malignant cells exhibited globally increased metabolic activity, with enrichment of pathways such as defects in biotin metabolism, biotin transport and metabolism, aspartate and asparagine metabolism, linoleic acid metabolism, sulfur metabolism and PP2A-related signaling. Biotin acts as a cofactor for key carboxylases involved in gluconeogenesis, fatty acid synthesis and amino acid metabolism, and perturbation of biotin utilization can impair the proliferation and invasiveness of glioma stem cells (Zastre et al., 2013; Yoon et al., 2021). Asparagine synthetase is often upregulated in cancer cells and supports survival under metabolic stress (Yuan et al., 2024), while circulating aspartate has been suggested as a potential risk factor for PCa (Lin et al., 2022). Linoleic-acid–derived eicosanoids generated via the 5-lipoxygenase (5-LOX) pathway can promote PCa cell growth (Moretti et al., 2004), and sulfur-containing amino acid metabolism contributes to redox homeostasis, antioxidant defenses and epigenetic regulation in cancer (Ward and DeNicola, 2019). PP2A is a central phosphatase that modulates multiple oncogenic signaling pathways, including EGFR, NF-κB and Wnt signaling (Nader et al., 2019). Although our data do not establish a direct mechanistic chain from PGAP3 to these pathways, the consistent enrichment of these metabolic programs in PGAP3-high malignant cells suggests that PGAP3 expression marks a metabolically reprogrammed tumor cell state that may favor PCa progression.

We also observed a robust relationship between PGAP3 expression and T-cell–related features in the PCa TME. Building on prior evidence that PGAP3 is associated with reduced CD8^+^ T-cell infiltration in breast cancer and that PGAP3 deficiency enhances T-cell receptor signaling (Raghupathy et al., 2015; Hao et al., 2023), our single-cell and spatial transcriptomic analyses demonstrated that PGAP3^+^ malignant regions show limited co-localization with cytotoxic T lymphocytes (CTLs) and are largely devoid of CTL infiltration. Bulk RNA-seq analyses in TCGA-PRAD further showed that high PGAP3 expression is associated with lower ESTIMATE scores, higher tumor purity and reduced ImmuneScores, indicating a less immune-infiltrated TME. Across cancers, TIMER 2.0 analyses revealed predominantly negative correlations between PGAP3 expression and CD8^+^ T-cell abundance, and TCellSI analysis indicated that PGAP3 expression is negatively associated with multiple T-cell functional states. Together, these observations support a strong negative association between PGAP3 expression and CD8^+^ T-cell infiltration and activity, although they do not prove that PGAP3 directly causes T-cell exclusion.

To explore potential mechanisms by which PGAP3-high tumor states might be linked to reduced CD8^+^ T-cell infiltration, we examined chemokines and immune-stimulating molecules. PGAP3-high tumors showed significantly lower expression of CXCL14, TNFSF13B and TNFSF18 compared with PGAP3-low tumors. CXCL14 has been associated with enhanced tumor-associated immune responses, increased cytotoxic CD8^+^ T-cell activity and greater T-cell infiltration (Kumar et al., 2022); TNFSF18 encodes GITR, which can augment CD8^+^ T-cell anti-tumor responses within the TME (Chu et al., 2019; Richards et al., 2019); and TNFSF13B expression has been linked to the composition of immune infiltrates, including a positive association with CD8^+^ T-cell presence (Ma et al., 2021). Reduced expression of these molecules in PGAP3-high tumors is therefore consistent with a microenvironment less permissive to CD8^+^ T-cell recruitment and activation. While our study cannot disentangle whether PGAP3 directly regulates these genes or whether they are co-regulated within broader tumor programs, the convergence of metabolic reprogramming, downregulation of immune-activating factors and CD8^+^ T-cell scarcity points to PGAP3-high tumor cells as a key component of the immune-cold phenotype in PCa.

Our functional assays complement the computational analyses by demonstrating that PGAP3 is upregulated in PCa cell lines and that siRNA-mediated PGAP3 knockdown suppresses proliferation, clonogenic growth and migration in C4-2 and DU145 cells. These observations are consistent with the proliferative and motility-related programs enriched in PGAP3-high malignant cells and support a role for PGAP3 in sustaining malignant phenotypes *in vitro*. However, more extensive functional studies using additional PCa models, orthogonal knockdown or knockout strategies and *in vivo* systems will be required to fully delineate the oncogenic functions of PGAP3 and their relationship to the immune microenvironment.

This study has several limitations. First, although we validated PGAP3 function using siRNA knockdown in PCa cell lines, rescue experiments and validation in immunocompetent *in vivo* models are still needed to better capture the *in situ* immune contexture. Second, our spatial quantification focuses on CTL/CD8^+^ signals; future work should extend the same framework to additional microenvironmental compartments (e.g., myeloid/TAM-like populations, Tregs, and stromal components) and incorporate interaction-based analyses. Finally, while PGAP3 shows consistent associations with metabolic programs and reduced CTL/CD8^+^ signals, the causal molecular links remain to be clarified.

## 5 Conclusion

By integrating MR-based genetic prioritization with bulk, single-cell and spatial transcriptomic analyses, comprehensive immune profiling, and *in vitro* functional validation, we identify PGAP3 as a tumor-intrinsic gene associated with metabolic reprogramming and a CTL/CD8^+^-low, immune-cold tumor microenvironment in PCa. Our findings support PGAP3 as a candidate biomarker of immune-cold tumors and motivate future mechanistic studies in immunocompetent systems. More broadly, the integrative framework used here provides a strategy for prioritizing and functionally annotating tumor-intrinsic programs linked to immune contexture in PCa and other malignancies.

## Data Availability

The datasets presented in this study can be found in online repositories. The names of the repository/repositories and accession number(s) can be found in the article/Supplementary Material.
