# Supplementary material for: Multi-omics integration identifies PGAP3 as a tumor-intrinsic factor associated with CD8+ T-cell exclusion in prostate cancer
Source: Front Mol Biosci. 2026 Mar 18;13:1791456. doi: 10.3389/fmolb.2026.1791456 (PMC13038432; doi:10.3389/fmolb.2026.1791456)
Supplement: Supplementary file 1 [file Supplementaryfile1.docx]

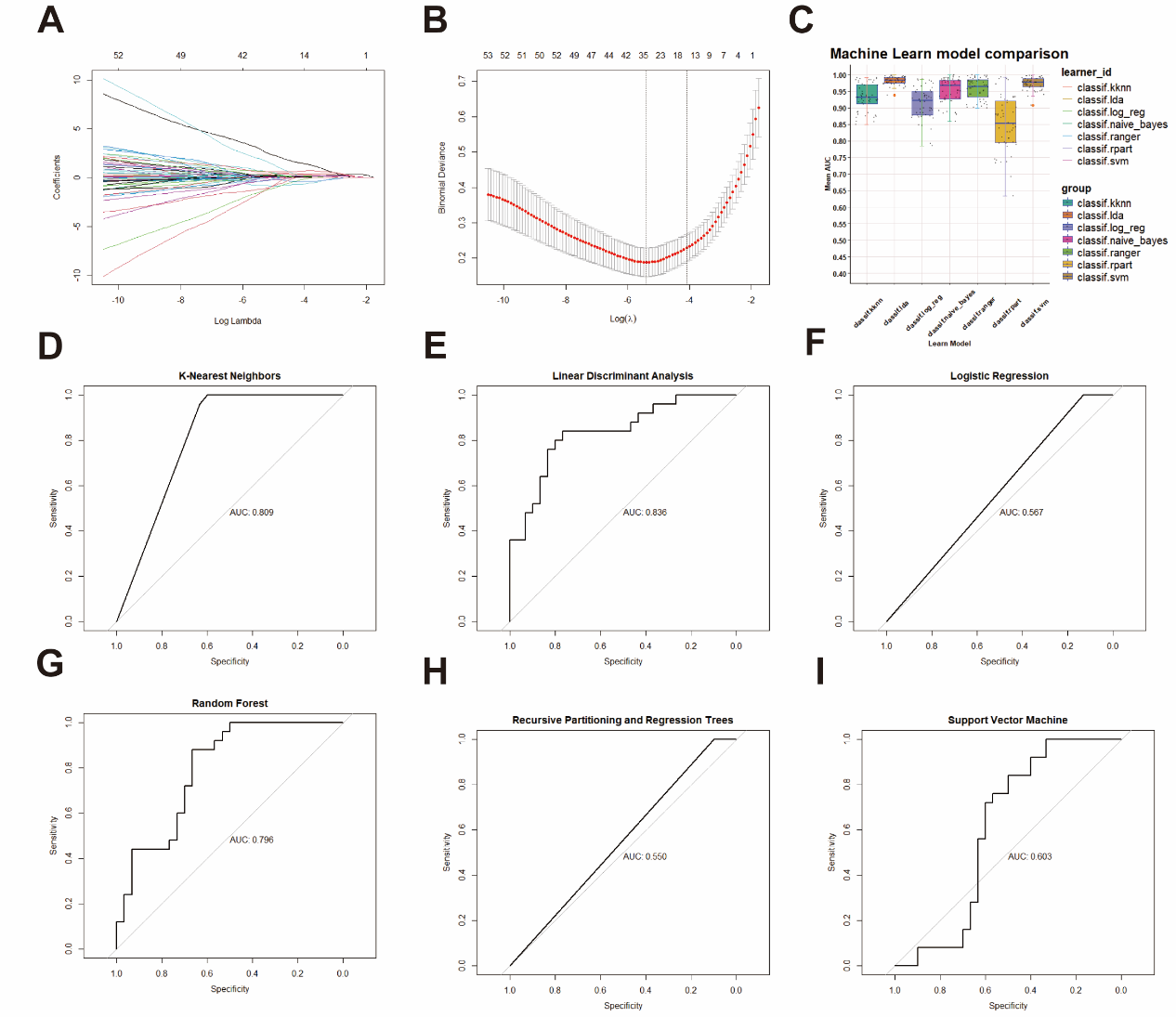


Fig S 1 Construction and Evaluation of binary response models. (A, B) cvfit and lambda curves showing the least absolute shrinkage and selection operator (LASSO) regression was performed with the minimum criteria. (C) Comparison of AUC values for 7 machine learning algorithms on the training set. (D-I) The AUC values of six machine learning algorithms on the validation set are as follows: K-Nearest Neighbors, Linear Discriminant Analysis, Logistic Regression, Random Forest, Recursive Partitioning and Regression Trees, and Support Vector Machine.


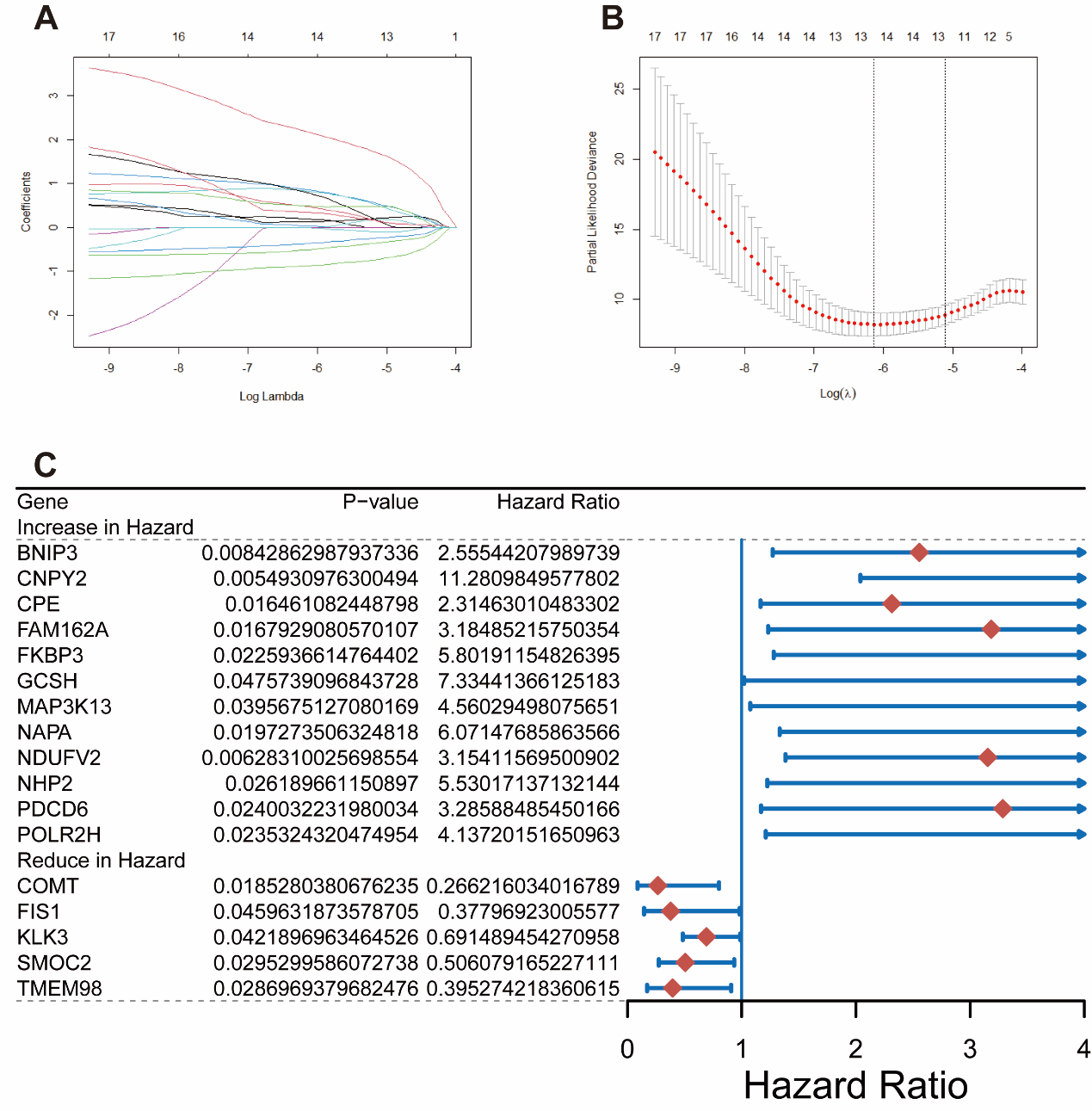


Fig S 2 Construction and Evaluation of prognostic model. (A, B) cvfit and lambda curves showing the LASSO regression was performed with the minimum criteria. (C) Forest plot of the genes involved in the model construction.


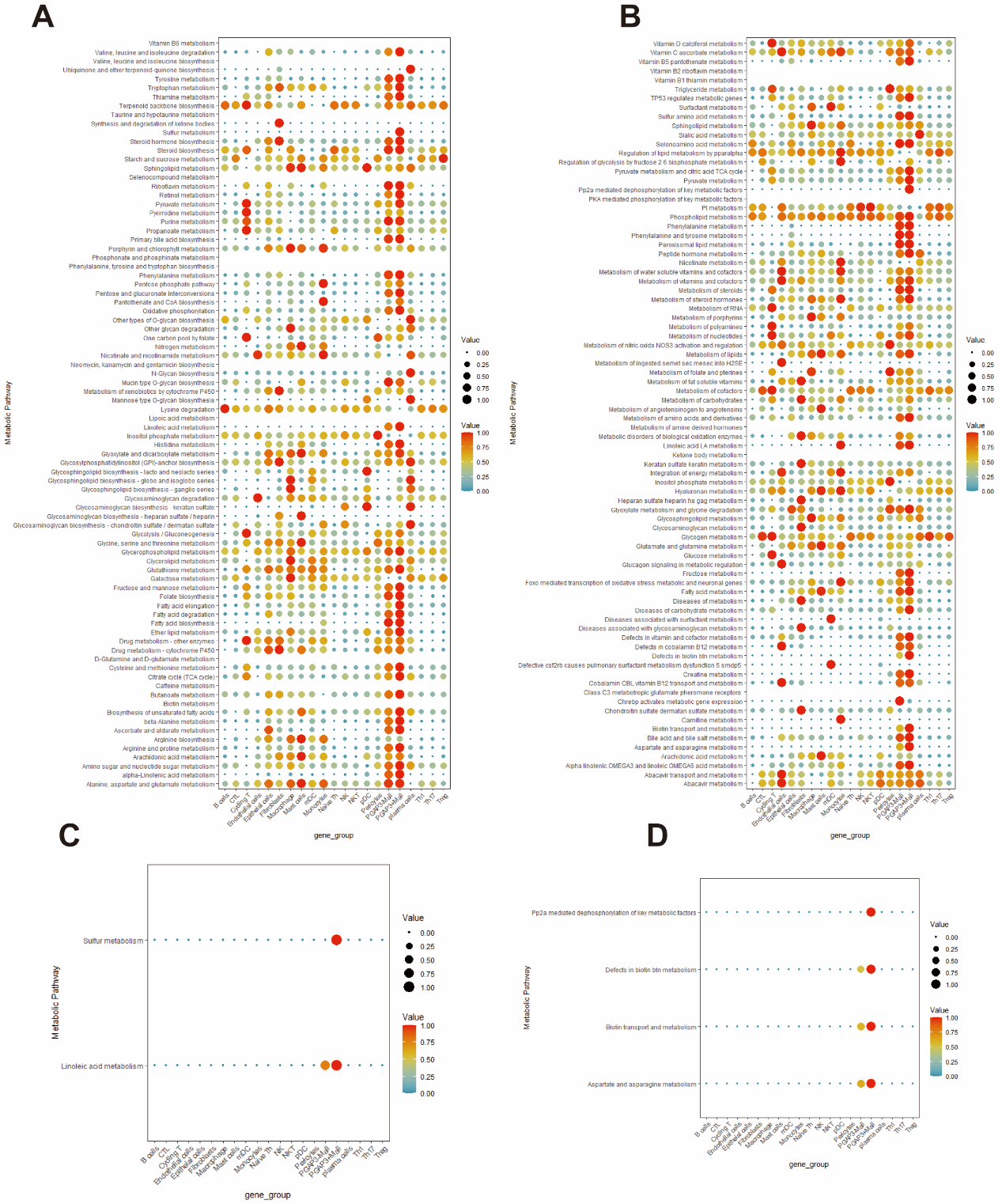


Fig S 3 The single-cell metabolic analysis of the 'PGAP3+ Malignant' subgroup. (A, B) Using scMetabolism to assess the metabolic activity of 85 KEGG pathways and 82 REACTOME terms, respectively. (C, D) Pathways specifically enriched in the 'PGAP3+ Malignant' subgroup, respectively among the 85 KEGG pathways and 82 REACTOME terms.


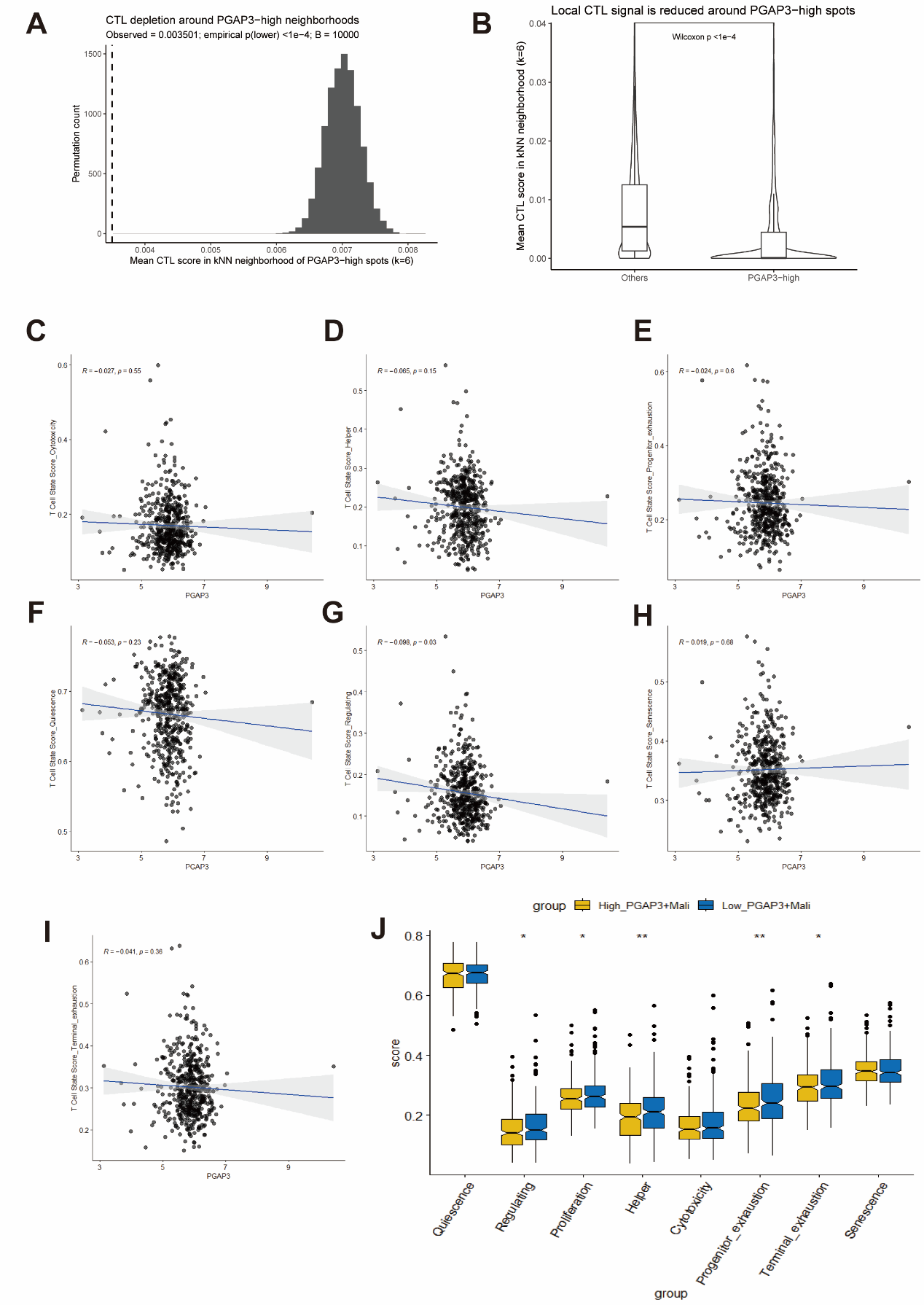


Fig S 4 Spatial quantification and T-cell state associations related to PGAP3. (A) Permutation-based kNN test (k = 6) showing reduced local CTL signal around PGAP3-high malignant spots (top 25% PGAP3+Mali; B = 10,000). Dashed line, observed statistic. (B) Permutation-based nearest-neighbor distance test showing increased spatial separation between PGAP3-high malignant spots and CTL-high spots (top 25% CTL; B = 10,000). Dashed line, observed statistic. (C-I) Scatter plots of the correlation between PGAP3 and the seven distinct states of T cells, namely Cytotoxicity, Helper, Progenitor_exhaustion, Quiescence, Regulating, Senescence, and Terminal_exhaustion, based on the TCellSI algorithm. (J) Box plots showing the differences in the proportions of 'PGAP3+ Malignant' groups compared to the eight different states of T cells.
